# Identification of sample donor by 24-plex short tandem repeat in a post-race equine plasma containing dexamethasone

**DOI:** 10.1186/2193-1801-3-94

**Published:** 2014-02-17

**Authors:** Jin-Wen Chen, Cornelius E Uboh, Lawrence R Soma, Youwen You, Zibin Jiang, Xiaoqing Li, Fuyu Guan, Ying Liu

**Affiliations:** University of Pennsylvania School of Veterinary Medicine, New Bolton Center Campus, 382 West Street Road, Kennett Square, PA 19348 USA; Pennsylvania Equine Toxicology & Research Center, Department of Chemistry, West Chester University, 220 E Rosedale Avenue, West Chester, PA 19382 USA

**Keywords:** Horse, Short tandem repeat, Plasma, Hair, Urine, Dexamethasone, Liquid chromatography tandem mass spectrometry

## Abstract

**Background:**

Animal sport such as horseracing is tainted with drug abuse as are human sports. Treatment of racehorses on race day with therapeutic medications in most cases is banned, and thus, it is essential to monitor the illicit use of drugs in the racing horse to maintain integrity of racing, ensure fair competition and protect the health, safety and welfare of the horse, jockeys and drivers. In the event of a dispute over the identity of the sample donor, if the regulator can provide evidence that the DNA genotype profile of the post-race sample matched that of the alleged donor, then the potential drug violation case might be easily resolved without legal challenges.

**Case description:**

We present a case study of a racehorse sample that tested positive for dexamethasone in a post-race plasma sample in Pennsylvania (PA) but the result was challenged by the trainer of the horse. Dexamethasone is a synthetic glucocorticoid widely used in the management of musculoskeletal problems in horses but its presence in the horse during competition is banned by the PA Racing Commissions. The presence of dexamethasone in the post-competition plasma sample was confirmed using liquid chromatography-tandem mass spectrometry. However, this finding was challenged by the trainer of the horse alleging that the post-race sample was not collected from his/her horse and thus petitioned the Commission to be absolved of any wrong-doing. To resolve the dispute, a DNA test was ordered by the PA Racing Commission to identify the correct donor of the dexamethasone positive sample. For this purpose, a 24-plex short tandem repeat analysis to detect 21 equine markers and three human markers was employed. The results indicated that all the samples tested had identical DNA profiles and thus, it was concluded that the samples were collected from the same horse and that the probability of drawing a false conclusion was approximately zero (1.5 × 10^-15^).

**Conclusions:**

The plasma sample confirmed for the presence of dexamethasone was collected from the alleged horse.

## Background

Animal sport such as horseracing is tainted with drug abuse as are human sports. Corticosteroids are steroidal anti-inflammatory drugs frequently used in racehorses to manage inflammation due to injury. It may mask pain to the extent that it would allow the horse to compete earlier than it is supposed to. The potential of a breakdown on the track by a horse that is prematurely racing is particularly dangerous because it puts the safety, health and welfare of the horse and lives of all other participants in the race at a great risk. To alleviate this potential problem, a rapid through-put screening, quantification and confirmation method was developed and put in place for the detection and confirmation of corticosteroids in post-race equine plasma samples (Luo et al., 2005). Treatment with therapeutic medications by the attending veterinarian to take care of a sick horse is allowed but it is against the regulation for a horse to compete while there is a quantifiable and confirmable concentration of any corticosteroid in the horse during competition.

In the event of a dispute over the identity of the sample donor, if the regulator can provide evidence of the DNA genotype profile of the post-race sample matching that of the alleged animal donor and of no other source of DNA present in the sample, then the potential drug violation case might be easily resolved without costly legal challenges. In response to the need for matching of a contested sample to the correct donor in the racing industry, we developed a novel 24-plex short tandem repeats (STR) analysis to detect 21 equine STR markers and three human markers to check for sample contamination by human DNA (Chen et al., 2010a).

This study presents a case of a post-race plasma sample that tested positive for the presence of dexamethasone but the result was challenged by the trainer of the horse claiming that the sample was not collected from his/her horse. When DNA profiles of the post-race samples (urine and plasma) and those of the alleged horse samples were compared using the novel 24-plex STR genotyping system, there was an indisputable match in the DNA profiles indicating that the post-race samples matched those of the alleged horse.

## Case description

All post-race samples were collected and witnessed by the respective trainer or representative. Sampling was approved by the Institutional Animal Care and Use Committee of the University of Pennsylvania. The samples were sealed according to the procedures that satisfy unbroken chain of custody as required by our Standard Operating Procedures and the accreditation Guidelines under ISO/IEC 17025. The samples were stored under proper conditions (urine and plasma at -20°C, whole blood and strands of hair at 4°C). Fifteen days after the post-race sample tested positive for dexamethasone, blood and strands of hair were collected from the alleged Standardbred horse by the attending veterinarian. Collection of the samples was witnessed by a PA Racing Commission representative and the horse trainer.

Routine drug screening analyses were performed on the post-race samples as previously described (You et al., 2009). The presence of dexamethasone in a post-race plasma sample was detected, quantified and confirmed using liquid chromatography-tandem mass spectrometry (LC-MS/MS) (Luo et al., 2005). Briefly, dexamethasone was recovered from plasma by liquid-liquid extraction using methyl tert-butyl ether. Analyte was separated on a Hypercarb column (2.1 × 30 mm) with acetonitrile:water:formic acid (95:5:0.5, v/v/v) mixture as the mobile phase and analyzed by positive electrospray ionization mode with selected reaction monitoring (SRM) on a triple quadrupole mass spectrometer (Finnigan TSQ Quantum Ultra with Accela Autosampler, Thermo Fisher Scientific, San Jose, CA, USA) (You et al., 2009). Retention time (t_R_) for dexamethasone was 1.08 ± 0.10 min. For screening and quantification analysis, SRM of *m/z* 393 → 355 transition was employed. Three ion transitions (*m/z* 393 → 355, *m/z* 393 → 337 and *m/z* 393 → 319) were monitored for confirmation. Similarity in ion intensity ratio and t_R_ were used as confirmation criteria. Deuterium-labeled dexamethasone (*d*_4_-dexamethasone, C/D/N ISOTOPES Inc., Quebec, Canada) was used as the internal standard (IS) for quantification. The linear calibration range was 0.25 - 100 ng/mL (r^2^ > 0.999). Limits of detection and quantification were 0.1 ng/mL and 0.25 ng/mL, respectively.

**Figure 1:**
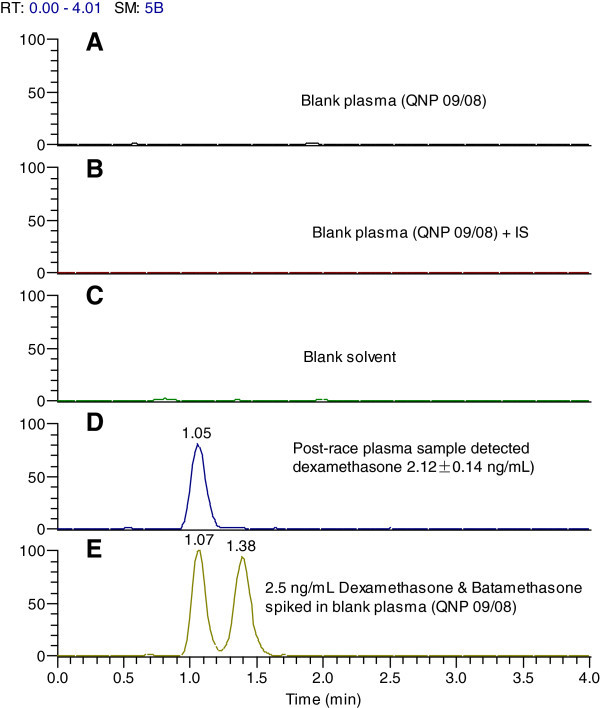
**LC-ESI** ^**(+)**^ **-MS/SRM chromatograms of dexamethasone in a post-race plasma, dexamethasone as calibrator and betamethasone as an isomer of dexamethasone.** Top three panels represent blank plasma **(A)**, blank plasma + IS **(B)** and solvent blank **(C)**, all of which were scanned for the presence of dexamethasone only at m/z 393 → 355. Panel **D** is dexamethasone detected in the post-race plasma sample collected from the alleged horse while panel **E** shows chromatographic resolution of dexamethasone (left) from betamethasone (right) spiked in blank plasma, extracted and analyzed. The t_R_ (1.05 min) of post-race plasma containing dexamethasone was similar to that for dexamethasone spiked plasma (calibrator) and the difference was within ± 0.1 min.

**Figure 2:**
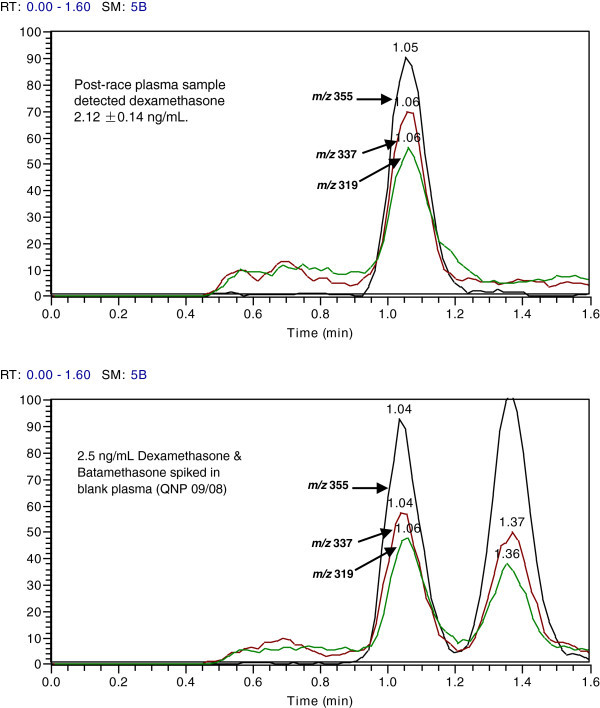
**Analyte confirmation by ion intensity ratio comparison of dexamethasone in a post-race plasma with that spiked in blank equine plasma.** Product ion intensity ratio of dexamethasone in the test sample (upper panel) compared with calibrator (lower panel, left) and with its isomer, betamethasone (lower panel, right) spiked in blank plasma, extracted and analyzed to ensure that the 3 criteria (similarity in product ion intensity ratio, similarity in 3 ion transitions and in t_R_) established for confirmation of the presence of dexamethasone in the test sample were satisfied. The difference in ion intensity ratio between the sample and the calibrator was < 20%.

Dexamethasone was detected, quantified (2.12 ± 0.14 ng/mL) and confirmed in one of the post-race plasma samples (Figure 1). Limits of detection, quantification and confirmation were 0.10, 0.25 and 0.50 ng/mL, respectively. Betamethasone is an isomer of dexamethasone and under normal analysis using liquid chromatography and mass spectrometry, dexamethasone cannot be distinguished from betamethasone because of similarity in chemical structure resulting in similarity in t_R_, mass spectrum and product ion spectrum. However, both compounds were chromatographically separated (Figure 1) (Luo et al., 2005). Based on this study, t_R_ of betamethasone was distinctly different from that of dexamethasone on the Hypercarb column and thus, these two drugs were chromatographically resolved (Figure 1E) from one another. In addition, the presence of dexamethasone in the post-race test sample was confirmed by chromatographic peak ion intensity ratio comparison and t_R_ (Figure 2).

Genomic DNA was isolated from blood, plasma, urine and hair root of the alleged horse using Genorise DNA Isolation Kits (Chen et al., 2010a) and quantified using agarose gel and OD_260_ using TotalLab software (Chen et al., 2009). Genomic DNA was also prepared from blood samples of two different horses that participated in the race as well as human hair for positive and negative control samples. A total of 2950 ng of DNA was recovered from 0.3 mL test plasma sample, 25 ng of DNA from 1 mL test urine, after the samples were refrigerated for 30 days. A total of 14.4 μg of DNA was obtained from 0.3 mL alleged blood sample while 56 ng of DNA was recovered from a single hair root; both of which were refrigerated for 24 h prior to DNA extraction.

Polymerase chain reaction (PCR) was conducted to detect STR as previously described (Chen et al., 2010a). Briefly, PCR primer pairs were designed to amplify twenty-one equine dinucleotide and three human tetranucleotide repeat markers for simultaneous detection of the STR markers in the suspect horse as well as in the control samples. One of each paired primers was labeled with one of three cyanine-based fluorescent dyes (WellRED D2-PA, D3-PA, and D4-PA; Beckman Coulter) to distinguish between markers. Twenty-one equine and three human STR markers were co-amplified in a reaction of 15 μL in a single tube in a thermocycler using Qiagen HotStar DNA Polymerase Master Kit.

**Figure 3:**
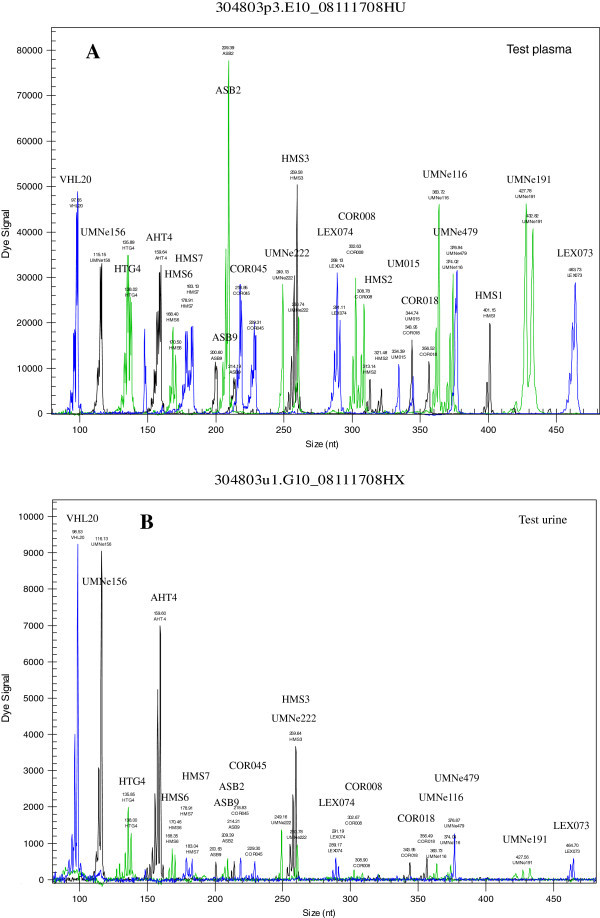
**Electropherograms of STR profiles from test plasma and test urine.** 24-plex STR profiling was conducted according to the method described previously (Chen et al., 2010a) and in the Case description. **(A)** 21 equine STR loci were detected in the test plasma. **(B)** 18 equine STR loci were detected in the test urine.

To acquire the STR profile of the alleged horse and of the control samples, fragment analysis of amplified STR products was performed (Chen et al., 2010a). Briefly, following initial verification of amplified STR fragments using 1.2% agarose gel, the visualized PCR products were further analyzed using capillary electrophoresis to determine the size of each STR marker. An aliquot (1 μL) of PCR products was analyzed on a CEQ8800 Genetic Analysis System (Beckman Coulter, Brea, CA, USA) and the size of each marker was estimated by automatic comparison with an internal size standard-600 using Fragment Analysis software. An allele was designated as the number of repeat unit plus 0.1 for partial repeat (one nucleotide) (Budowle et al., 2005). A complete DNA profile was obtained from the alleged blood and hair root samples and the post-race plasma sample as indicated in Figure 3A and Table 1. STR profiles containing 21 equine STR markers were identical in the samples (blood and hair) collected from the alleged horse that had been sanctioned by the PA Racing Commission and that of the post-race plasma sample, indicating that the post-race plasma was obtained from the alleged horse. Due to insufficient quantity or quality of sample, 18 out of 21 markers were detected in the post-race urine sample and the 18 markers also matched those of the subsequent samples collected from the alleged horse (Figure 3B); this result further verified the origin of all the samples analyzed. As expected, none of the human DNA markers was detected in all the samples analyzed, indicating that none of the equine samples analyzed was contaminated by human DNA (data not shown).

**Table 1:** **Comparison of genotypes between suspect and test samples**

	VHL20	UMNe156	HTG4	AHT4	HMS6	HMS7	ASB9	ASB2	COR045	UMNe222	HMS3
Test plasma	18/18	21/21	19/20	33/33	19/20	20/22	14/21	22/22	15/21	10/16	25/25
Test urine	18/18	21/21	19/20	33/33	19/20	20/22	14/21	22/22	15/21	10/16	25/25
Suspect blood	18/18	21/21	19/20	33/33	19/20	20/22	14/21	22/22	15/21	10/16	25/25
Suspect hair	18/18	21/21	19/20	33/33	19/20	20/22	14/21	22/22	15/21	10/16	25/25
	**LEX074**	**COR008**	**HMS2**	**UM015**	**COR018**	**UMNe116**	**UMNe479**	**HMS1**	**UMNe191**	**LEX073**	
Test plasma	22/23	21/24	18/22	13/19	10.1/17	18/23	24/24	19/19	15/17	34/34	
Test urine	22/23	21/24	ND	ND	10.1/17	18/23	24/24	ND	15/17	34/34	
Suspect blood	22/23	21/24	18/22	13/19	10.1/17	18/23	24/24	19/19	15/17	34/34	
Suspect hair	22/23	21/24	18/22	13/19	10.1/17	18/23	24/24	19/19	15/17	34/34	

ND: not detected.

In the present study, three STRs (HMS2, UM015 and HMS1) were not detected in the test urine, indicating typing deficiency of these STRs as well as limitation of the urine sample. It is not surprising that it is difficult to show a complete STR profile in urine sample due to the nature of the sample and especially after a substantial period of storage (Chen et al., 2009). In an independent study conducted, two random horses showed different STR profiles and the profile of human hair did not show presence of the horse STR fragments (data not shown). Again, these results indicated that the findings for the alleged horse were corroborated and that the STR genotyping method used is valid for individual sample discrimination.

**Figure 4:**
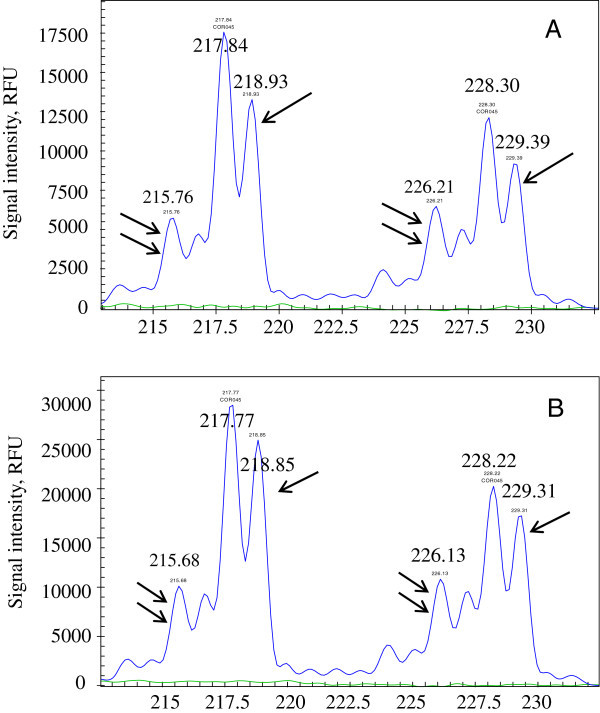
**Electropherograms of allelic, plus A and stutter peaks at locus COR045.** Alleic, plus A and stutter peaks were detected at locus COR045. The size of all peaks was labeled with the length of the amplified DNA fragment as determined by Fragment Analysis software (v. 3.2.42; Beckman Coulter). **(A)** COR045 profile for the suspect horse. **(B)** COR045 profile for the post-race plasma.

Plus A products of the amplified STRs were sequenced using GenomeLab™ Dye Terminator Cycle Sequencing Quick Start Kit (Beckman Coulter, Brea, CA, USA) (Chen et al., 2010a). A single PCR product of plus A signal was isolated from 6% polyacrylamide gel prior to sequencing. Plus A peak was present in three loci including ASB9, HMS7 and COR045. These plus A peaks were considered artificial peaks that are frequently identified as an STR fragment in addition to a non-template adenosine at the 3′ end of the fragment. The peak was one nucleotide longer than the real allelic peak and was not considered an allele. For instance, two plus A peaks were detected at locus COR045 in post-race samples and in those collected from the alleged horse. In the blood sample collected from the suspect horse, 218.93 and 229.39 were considered plus A peaks (Figure 4A, arrows). In the post-race plasma, 218.85 and 229.31 were also considered plus A peaks (Figure 4B, arrows). These two samples showed an identical peak pattern at locus COR045 although plus A peak was presented in both alleles. Similar patterns were observed at loci ASB9 and HMS7 (data not shown). The non-template adenosine was confirmed by sequencing (GenBank accession #KJ021053, KJ021054, KJ021055, KJ021056, KJ021057 and KJ021058) and was located at the 3′end of the allelic fragment. Plus A peaks were smaller than their counterpart, allelic peaks (56–79%).

Stutter activity was observed in most STR loci and peak area ratios were 0.2-0.4, and were in agreement with those previously reported (Chen et al., 2010a). For instance, stutter activity was observed at locus COR045 (Figure 4), the suspect horse and the post-race plasma samples showed an identical stutter pattern including stutter peaks of approximately 216 bp and 226 bp (Figure 4 double arrows). The presence of the stutter activity did not influence interpretation of the STR results since it was common and the stutter peaks were identical in the two samples.

Using allele frequency as previously defined, random match probability (RMP) was calculated as a product of genotype frequency for identical alleles between samples using 13 independent loci (Chen et al., 2010a, 2010b; Fung et al., 2006). The computed probability is a measure of matching probability between two random samples, and thus, the smaller RMP indicates higher probability of a match. RMP reflects the probability of a random animal as a true donor and was calculated as the probability of accepting a random horse being a culprit subject. RMP in the present case study was 1.5 × 10^-15^ for the samples from the alleged horse and 1.6 × 10^-11^ for the urine sample vs samples from the alleged horse. Taken together, this result indicated that the probability of drawing a false conclusion regarding this case was nearly zero. Alleles detected in the alleged and post-race samples were also detected in a previous study (Chen et al., 2010a). However, allele 14 at locus ASB9 and allele 15 at locus COR045 were not previously reported (Table 2) although they were detected in other case studies (not published). In the previous study, the use of 13 independent loci to calculate RMP was validated. Those loci were located at different chromosome and showed no linkage disequilibrium. The remaining eight loci were not used in the estimation of the RMP because of synteny. Among 21 loci, UMNe156, ASB2, UMNe222, HMS1 are syntenic (chromosome 15); HTG4, COR008 and HMS3 are syntenic (chromosome 9); AHT4 and LEX074 are syntenic (chromosome 24); ASB9, COR045 and HMS2 are syntenic (chromosome 10).

**Table 2:** **Allele frequency for alleles detected in the suspect Standardbred pacer horse and post-race samples (n = 171)**

STR	Allele	Frequency	Allele	Frequency
VHL20*	18	0.377	NA	NA
UMNe156	21	0.544	NA	NA
HTG4	19	0.108	20	0.099
AHT4*	33	0.474	NA	NA
HMS6*	19	0.447	20	0.307
HMS7*	20	0.377	22	0.518
ASB9	14	0. 01	21	0.011
ASB2*	22	0.205	NA	NA
COR045	15	0.01	21	0.146
UMNe222	10	0.175	16	0.693
HMS3*	25	0.272	NA	NA
LEX074	22	0.240	23	0.301
COR008	21	0.129	24	0.477
HMS2*	18	0.386	22	0.082
UM015*	13	0.123	19	0.006
COR018*	10.1	0.064	17	0.594
UMNe116*	18	0.211	23	0.155
UMNe479*	24	0.711	NA	NA
HMS1	19	0.345	NA	NA
UMNe191*	15	0.020	17	0.243
LEX073*	34	0.249	NA	NA

*Allele frequency was based on a database for Standardbred horses (n = 171) (Chen et al. 2010a) and random match probability was estimated over 13 independent loci. NA = not applicable.

## Discussion and evaluation

The presence of dexamethasone in the post-race plasma sample from the alleged horse was detected, confirmed, quantified and reported to the PA Racing Commission. When the trainer was informed of the violation, he/she immediately claimed that the sample could not have been collected from his/her horse because he/she did not administer the drug to the horse and did not authorize anyone to do so. In order for the Commission to resolve the controversy, DNA test on the samples was ordered. Results obtained indicated that all the samples tested were obtained from the same horse.

Dinucleotide-based STR profiling for individual discrimination has been successfully employed in the horse (Chen et al., 2010a; van de Goor et al., 2010). However, precision in calling of alleles using dinucleotide STR loci in forensic science presents a serious limitation. In human forensics, dinucleotide STR loci are no longer used because of the frequent occurrence of stuttering and the difficulties with precision in calling the true alleles, especially when the allele span difference is one repeat unit. Similar to our system, a previous proposal for standardization in forensic equine DNA typing used 17 equine-specific STR loci, but only 13 independent loci can be used for calculation of RMP (van de Goor et al., 2010). However, a comprehensive study showing a match of post-race samples with that in which detection, quantification and confirmation of a banned substance in a post-race equine plasma sample had not previously been reported (Tobe et al., 2006). The result obtained from the novel 24-plex STR genotyping system unambiguously demonstrated that the post-race equine plasma sample that tested positive for the presence of dexamethsaone was collected from the same horse (Table 1).

Plus A peak is a non-specific artifact during PCR amplification of the STR markers and was common (Brownstein et al., 1996; Clark, 1988) but had not been reported in the equine (Bowling et al., 1997; van de Goor et al., 2010, 2011). Taq DNA polymerase showed capability of promoting non-template addition of adenosine to the 3′ end of PCR–amplified products (Brownstein et al., 1996). In comparison with stutter peaks (Chen et al., 2010a, 1996; Gill et al., 1997), plus A signals were easily detected by Fragment Analysis Software (v. 3.2.42) incorporated in the Genetic Analysis System (Beckman Coulter) and could be easily excluded during data processing and genotype determination (Chen et al., 2010a). Although dinucleotide STR loci are still being used in equine STR profiling, exploring other means such as equine-specific tetranucleotide STR loci, single nucleotide polymorphism or analysis of mitochondrial DNA may be an alternative or a better approach for interpreting and validating genotyping result (Walsh et al., 1996; Breen et al., 1994; Kakoi et al., 2013; Xu et al., 2011; Allen et al., 1998).

Following storage, the integrity of DNA in urine sample was shown to decline thus, reducing the chances of detecting alleles (Chen et al., 2009) but after a month of storage at 4°C, most STRs were still detected in the samples in the present study. It is evident that the 24-plex STR typing system is capable of revealing a complete STR profile with 21 markers from 30-day old refrigerated plasma sample although it had been reported that long-term storage could decrease the quality and quantity of DNA sample (Chen et al., 2009). Identity of the post-race and alleged samples in this study was confirmed by an independent laboratory in California that analyzed the “B” samples using thirteen DNA markers (personal communication with the PA Racing Commission). Like other genotyping systems (Kakoi et al., 2013; van de Goor et al., 2010), the present system is not perfect but it demonstrates a remarkable success on a number of cases including dry blood stains retained in a suspect syringe (Chen et al., 2010b) and is accredited under ISO 17025 Guidelines as a standard DNA test procedure. This is an intriguing study with results showing that on race day, an unauthorized drug, dexamethsaone, was detected in a post-race plasma sample and that the origin of the sample was correctly identified. Sample identification by DNA provided corroborated evidence that the samples analyzed were obtained from the same horse.

## Conclusion

The post-race plasma sample contained dexamethasone as confirmed by LC/MS/MS and STR profile of the sample matched that of the suspect horse.

## Abbreviations

DNA: Deoxyribonucleic acid
ESI: Electrospray ionization
IS: Internal standard
LC-MS/MS: Liquid chromatography tandem mass spectrometry
PCR: Polymerase chain reaction
RMP: Random match probability
SRM: Selected reaction monitoring
STR: Short tandem repeat.
